# A High‐Resolution Ternary Model Demonstrates How PEGylated 2D Nanomaterial Stimulates Integrin *α*_v_ *β*_8_ on Cell Membrane

**DOI:** 10.1002/advs.202004506

**Published:** 2021-03-23

**Authors:** Xiao Zhang, Zhaowen Ding, Guanghui Ma, Wei Wei

**Affiliations:** ^1^ State Key Laboratory of Biochemical Engineering Institute of Process Engineering, Chinese Academy of Sciences No. 1 Bei‐Er‐Tiao, Zhong‐Guan‐Cun, Haidian District Beijing 100190 P. R. China; ^2^ School of Chemical Engineering University of Chinese Academy of Sciences No. 19A Yuquan Road Beijing 100049 P. R. China

**Keywords:** conformational changes, integrin activation, molecular dynamics simulations, passivated 2D nanomaterials, ternary model

## Abstract

Bio–nano interfaces are integral to all applications of nanomaterials in biomedicine. In addition to peptide‐ligand‐functionalized nanomaterials, passivation on 2D nanomaterials has emerged as a new regulatory factor for integrin activation. However, the mechanisms underlying such ligand‐independent processes are poorly understood. Here, using graphene oxide passivated with polyethylene glycol (GO‐PEG) as a test bed, a ternary simulation model is constructed that also includes a membrane and both subunits of integrin *α*_v_ *β*_8_ to characterize GO‐PEG‐mediated integrin activation on the cell membrane in a ligand‐independent manner. Combined with the experimental findings, production simulations of the ternary model show a three‐phase mechanotransduction process in the vertical interaction mode. Specifically, GO‐PEG first induces lipid aggregation‐mediated integrin proximity, followed by transmembrane domain rotation and separation, leading to the extension and activation of extracellular domains. Thus, this study presents a complete picture of the interaction between passivated 2D nanomaterials and cell membranes to mediate integrin activation, and provides insights into the potential de novo design and rational use of novel desirable nanomaterials at diverse bio–nano interfaces.

## Graphical abstract

Passivated 2D nanomaterials have emerged as new regulatory factors for integrin activation in a ligand‐independent manner. Taking PEGylated graphene oxide as a test bed, it first induces lipid aggregation‐mediated integrin *α*_v_ *β*_8_ proximity, followed by transmembrane domain rotation and separation, leading to the extension and activation of extracellular domains. It provides a clue for the design and use of nanomaterials.

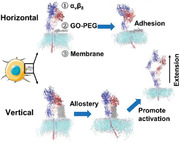

## 1 Introduction

Bio–nano interfaces are established when nanomaterials interact with biological components, such as cells, proteins, and membranes,^[^ ^1^, ^2^ ^]^ and comprise a series of time‐dependent dynamic interactions, which dominate the physicochemical reactions, kinetics, and thermodynamic exchanges between the surfaces of nanomaterials and biological components.^[^ ^3^ ^]^ These interactions are integral to biological processes such as cellular contact, ligand recognition and activation, intracellular signaling transduction, and cell behavior, which are closely related to normal physiological responses and human health.^[^ ^4^ ^]^ Accordingly, an understanding of these processes should enable further manipulations of bio–nano interfacial interactions to advance the biomedical application of nanomaterials.

At the bio–nano interface, integrins are known to mediate interactions between cells and nanomaterials.^[^ ^5^, ^6^ ^]^ Multiple studies have shown that nanomaterials bound to proteinaceous ligands (such as RGD peptides)^[^ ^7^, ^8^ ^]^ can activate integrins by specifically binding to the ligand recognition sites in their extracellular (EC) domains, resulting in conformational changes and *α* and *β* subunit transmembrane (TM) domain separation.^[^ ^9^, ^10^, ^11^, ^12^ ^]^ Moreover, the mechanosensitive nature of integrins aids their activation via direct physical force.^[^ ^13^, ^14^, ^15^ ^]^ Although such discoveries suggest the possibility of mechano‐stimulating cells based on rational nanomaterial design, the mechanism(s) underlying this ligand‐independent integrin activation are not well understood. This is partially due to the inability to monitor dynamic interactions using methods based on the co‐crystallization of proteins with nanomaterials (e.g., nuclear magnetic resonance, X‐ray diffraction, and cryo‐electron microscopy). To overcome this limitation, several molecular dynamics simulations have been employed to explore the dynamic interactions that occur at the bio–nano interface, even with atom‐scale resolution. However, present studies only modeled binary systems comprising a nanomaterial with either a membrane or a protein.^[^ ^16^, ^17^, ^18^, ^19^ ^]^ Hence, such binary systems fail to reflect the ternary interactions that occur among nanomaterials, membrane lipids, and integrin subunits.

We have previously shown that the 2D graphene oxide nanosheet chemically passivated with polyethylene glycol (GO‐PEG) interacts with the plasma membrane of macrophages and further activates the integrin *α*_v_ *β*_8_‐mediated mechanical signaling pathway for immunoactivation.^[^ ^20^ ^]^ This discovery revealed an experimental test bed for investigating bio–nano interfaces for ligand‐independent integrin signaling activation. In this study, we sought to understand the mechanism of PEGylated 2D nanomaterial‐mediated integrin activation in a ligand‐independent manner combining molecular dynamics simulations and experimental verifications. Specifically, we developed a ternary simulation model and investigated the atomic interactions of GO‐PEG, membranes, and integrin *α*_v_ *β*_8_ both horizontally and vertically. Vertical production simulations of the ternary model showed a three‐phase mechanotransduction process, along with experiments: Phase I: GO‐PEG mediated membrane lipid aggregation and drove the proximity of integrin *α*_v_ *β*_8_; Phase II: the TM domains of *α*_v_ *β*_8_ rotated and separated due to GO‐PEG stimulation; Phase III: the EC domains of *α*_v_ *β*_8_ responded to TM conformational changes for *α*_v_ *β*_8_ extension and activation. Thus, our study not only presents a complete picture of the interactions between 2D nanomaterials and cell membranes that mediate integrin activation based on the ternary model, but also provides insights into the rational design and use of new nanomaterials to obtain desired outcomes at diverse bio–nano interfaces.

## 2 Results

### 2.1 Observation of Bio–Nano Interfacial Interactions and Construction of Ternary Model

**Figure 1:**
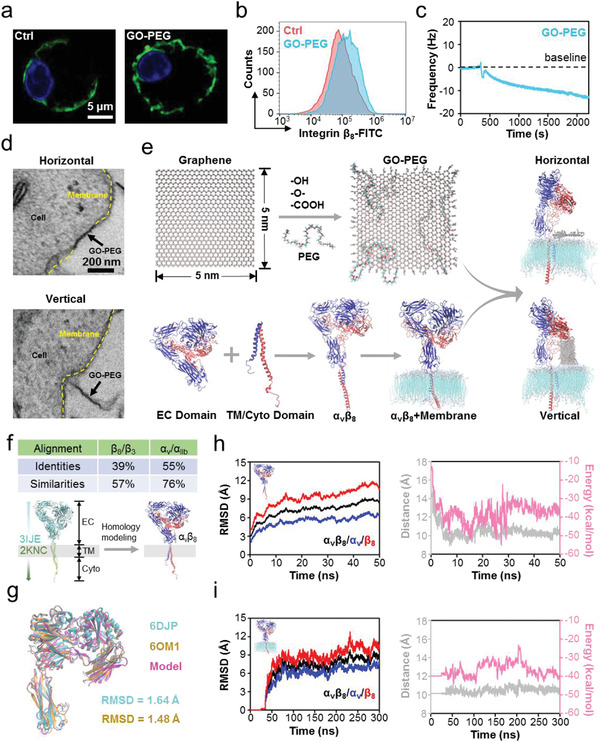
Nano–bio interface interaction and the corresponding ternary coupling model comprising PEGylated graphene oxide (GO‐PEG), integrin, and membrane. a) Confocal laser scanning microscope images of integrin *β*_8_ (green) on peritoneal macrophages with and without GO‐PEG stimulation. The expression of integrin *β*_8_ is higher after GO‐PEG stimulation. b) Flow cytometry analysis of integrin *β*_8_ on peritoneal macrophages with and without GO‐PEG stimulation. It also proves the higher expression of integrin *β*_8_. c) QCM curves of GO‐PEG flowing across a macrophage membrane spin‐coated on the chip to detect the mechanical interaction of nano–bio interface. The downward frequency reflects an increased quality on the chip in response to the bound GO‐PEG. d) Nano–bio interface interaction mode of GO‐PEG and a macrophage imaged via TEM. Upper panel, horizontal mode; lower panel, vertical mode. e) Construction of the ternary model comprising GO‐PEG, *α*_v_ *β*_8_, and a POPC bilayer. The GO‐PEG and POPC bilayer models were built using VMD plugins; the *α*_v_ *β*_8_ model was built using a standard homology modeling method. The interaction mode between GO‐PEG and the POPC bilayer includes horizontal and vertical modes. Blue, *α* subunit of *α*_v_ *β*_8_; Red, *β* subunit of *α*_v_ *β*_8_. f) Sequence alignment of the primary sequences between *α*_v_ *β*_8_ and its templates, and schematic diagram of homology modeling. g) Comparison between homology modeling‐based *α*_v_ *β*_8_ model and *α*_v_ *β*_8_ crystal structure. Pink, homology modeling model; Cyan, crystal structure (PDB ID: 6DJP); Orange, crystal structure (PDB ID: 6OM1). The RMSD is calculated by superimposing the protein backbone atoms; the low RMSD values validate the reliability of homology modeling. h) *α*_v_ *β*_8_ model stability during equilibrium simulation. Left panel: RMSD calculations for *α*_v_ *β*_8_, *α* subunit, and *β* subunit. Right panel: centroid distance and noncovalent interaction energy of the TM domains. These parameters were stable after 50 ns equilibrium simulation, indicating that the terminal structure can be utilized in the subsequent simulations on membrane. i) *α*_v_ *β*_8_ stability on membrane during equilibrium simulation. Left panel: RMSD calculations for *α*_v_ *β*_8_, *α* subunit, and *β* subunit. Right panel: centroid distance and noncovalent interaction energy of TM domains. These parameters were stable after 300 ns equilibrium simulation, indicating that the terminal structure can be utilized in production simulation.

It has been suggested that PEG‐functionalized nanomaterials elicit less immune responses than their pristine counterparts. We have previously shown a contradictory phenomenon, i.e., GO‐PEG eliciting strong immunological responses from primary macrophages, despite not being internalized. In‐depth investigation revealed a novel intracellular signaling pathway initiating from integrin *α*_v_ *β*_8_, while the detailed bio–nano interfacial interaction was unclear.^[^ ^20^ ^]^ In the present study, we successfully prepared GO‐PEG (Figure S1, Supporting Information) according to our previous protocol and used confocal imaging to profile integrin *β*_8_ (an essential part of integrin *α*_v_ *β*_8_) (Figure S2, Supporting Information) distribution on primary macrophages. This showed that 10 µg mL^−1^ GO‐PEG‐treated macrophages had a significantly larger integrin *β*_8_ area compared to that of the untreated control group and GO‐treated group at 24 h (**Figure** **1a** and Figure S3a, Supporting Information), suggesting that the increased *β*_8_ played an important role in the immunoactivation. We also used flow cytometry and western blotting to confirm that GO‐PEG increased the surface expression and overall accumulation level of integrin *β*_8_, respectively (Figure 1b and Figure S3b,c, Supporting Information).

We explored the bio–nano interfacial interactions between GO‐PEG and the macrophage plasma membrane using quartz crystal microbalance (QCM). The oscillation frequency (∆*F*) of a chip spin‐coated with the extracted cell membranes (collected from primary macrophages, and containing membrane proteins) substantially increased after injecting GO‐PEG, supporting the interaction between GO‐PEG and plasma membrane (Figure 1c). To directly observe the bio–nano interface, we also analyzed the GO‐PEG/macrophage samples using transmission electron microscopy (TEM) after co‐incubation for 24 h. The TEM images displayed interfacial interactions, which were mainly classified into two modes: in the first mode, GO‐PEG was parallel adhesion on the macrophage, horizontally interacting with the plasma membrane (termed as the “horizontal” mode); in the second mode, GO‐PEG was perpendicular entry on the macrophage, vertically interacting with the plasma membrane (termed as the “vertical” mode) (Figure 1d and Figure S4, Supporting Information).

To further clarify the distinct horizontal and vertical interfacial interaction modes between GO‐PEG and macrophages, we performed extensive molecular dynamics simulations. We computationally generated three models that enable ternary interaction simulations: a GO‐PEG nanosheet, a 1‐palmitoyl‐2‐oleoyl‐*sn*‐glycero‐3‐phosphocholine (POPC) lipid membrane, and an integrin *α*_v_ *β*_8_, for developing robust models that represent physiological contexts realistically. The complete process used for preparing each model has been described in the Experimental section. Briefly, GO‐PEG was produced using the VMD nanotube builder plugin, and the POPC membrane bilayer fragment was generated using the VMD membrane builder plugin (according to a standard procedure).^[^ ^21^ ^]^ To construct integrin *α*_v_ *β*_8_, we used a standard homology modeling method and previously published structures in the PDB database as templates for modeling,^[^ ^22^ ^]^ specifically including the *α*_v_ *β*_3_ EC domains (PDB ID: 3IJE), as well as both the TM and intracellular domains of *α*_IIb_ *β*_3_ (PDB ID: 2KNC). After a series of equilibrium simulations to ensure that these models could reach a stable state, they were assembled as a ternary simulation model and production simulations were performed (Figure 1e).

Since the complete integrin *α*_v_ *β*_8_ structure has not been published in the PDB database till date, we used homology modeling to construct the *α*_v_ *β*_8_ model. To verify its reliability, we aligned the primary sequences between *α*_v_ *β*_8_ and the above‐mentioned templates using BLAST plugin in the NCBI database. The results showed that the *β*_8_ subunit sequence was 39% identical and 57% similar to the *β*_3_ subunit sequence, and the TM and intracellular domains of the *α*_v_ subunit were 55% identical and 76% similar to those of the *α*_IIb_ subunit (Figure 1f). These support the protein homology between our model and templates and their physiological relevance.^[^ ^23^ ^]^ Meanwhile, the constructed *α*_v_ *β*_8_ model was consistent with the published partial integrin *α*_v_ *β*_8_ structure (PDB ID: 6DJP, 6OM1),^[^ ^24^, ^25^ ^]^ with a root mean square deviation (RMSD) value for the structural comparison being 1.64 and 1.48 Å (Figure 1g).

To verify the structural stability of the constructed *α*_v_ *β*_8_ model, we also conducted equilibrium simulations for the initial structure of the newly built integrin *α*_v_ *β*_8_. We separately calculated the RMSD values for the *α* subunit, *β* subunit, and total *α*_v_ *β*_8_, which revealed that the *α*_v_ *β*_8_ structure was stable after 50 ns equilibrium simulation. The centroid distance and interaction energy of the TM domains, which is known to be essential for integrin activation, also reached a steady state (Figure 1h). These data indicate that the terminal conformational state of integrin *α*_v_ *β*_8_ after 50 ns equilibrium simulation may be used for subsequent simulations. We then combined the balanced *α*_v_ *β*_8_ structure with the POPC membrane to investigate the binary simulation system. The RMSD values were stable after 300 ns equilibrium simulation. The *α*_v_ *β*_8_ TM domains were stable, with 10 Å centroid distance and −40 kcal mol^−1^ interaction energy (Figure 1i). All these results demonstrate the reliability and stability of the constructed *α*_v_ *β*_8_ for ternary production simulations.

### 2.2 GO‐PEG Mediates POPC Membrane Lipid Aggregation in the Vertical Mode

**Figure 2:**
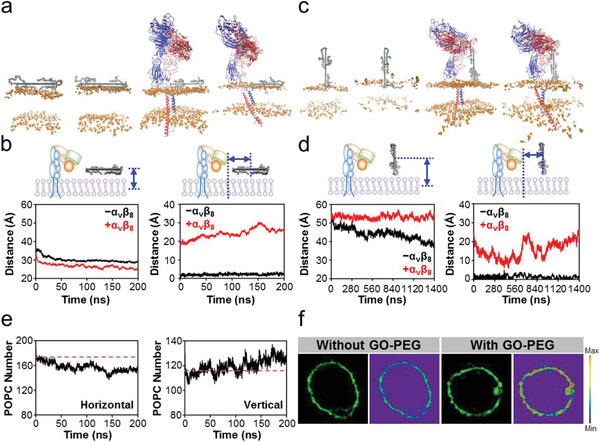
Effects of GO‐PEG on POPC membrane in the horizontal and vertical mode of ternary model. a) Structure at the initial and terminal moments of production simulation in the horizontal mode. GO‐PEG is placed in the same horizontal position. Blue, *α*_v_ subunit; Red, *β*_8_ subunit; Orange, head piece of POPC lipids; Other, GO‐PEG. The membrane is simplified as the area between the head of POPC lipids to facilitate the observation of changes in the membrane. b) Vertical‐ and horizontal‐centroid distances between GO‐PEG and POPC membrane in the horizontal mode. GO‐PEG is adsorbed on POPC membrane surface and maintains a relatively stable state. c) Structure at the initial and terminal moments of production simulation in the vertical mode. GO‐PEG is placed in the same horizontal position. The legend descriptions refer to those in (a). d) Vertical‐ and horizontal‐centroid distances between GO‐PEG and the POPC bilayer. *α*_v_ *β*_8_ inhibits the extraction of vertical GO‐PEG‐induced POPC membrane lipids and mediates a three‐step horizontal motion of POPC membrane lipids. e) Number of POPC membrane lipids within 4 nm of GO‐PEG in the production simulation of ternary model. Left panel: horizontal mode; Right panel: vertical mode. POPC membrane lipids clustered with GO‐PEG in the vertical mode. f) Representative fluorescence imaging on lipid aggregation of macrophage with and without GO‐PEG stimulation. The membrane lipids were labeled with lipophilic *β*‐BODIPY dye.

Next, we conducted production simulations with the models to dissect the interaction mechanisms of GO‐PEG with the POPC membrane either with or without integrin *α*_v_ *β*_8_. In the horizontal mode, GO‐PEG first diffused freely on the membrane surface, bound rapidly to the POPC membrane, and reached a stable state, regardless of *α*_v_ *β*_8_ presence (**Figure** **2a**). Both the horizontal‐ and vertical‐centroid distances between the GO‐PEG and POPC membranes indicated this interaction phenomenon (Figure 2b). In the vertical mode, GO‐PEG interacted with the POPC membrane and extracted POPC lipids from the membrane to its surface. Eventually, GO‐PEG was partially wrapped by the POPC membrane (Figure 2c), which was consistent with our previously reported results.^[^ ^20^ ^]^ The visible decline of the vertical‐centroid distance between the GO‐PEG and POPC membranes (from 52 to 38 Å) validated this result (Figure 2d).

However, in the presence of *α*_v_ *β*_8_, this lipid extraction phenomenon was suppressed (Figure 2c). The vertical‐centroid distance between the GO‐PEG and POPC membranes was stable at about 53 Å during the production simulation of the ternary model (Figure 2d). These data for the vertical mode might be attributed to the enhanced stability of the POPC membrane in the presence of *α*_v_ *β*_8_. Interestingly, in the absence of *α*_v_ *β*_8_, the horizontal‐centroid distance between GO‐PEG and the POPC membrane was persistently consistent around 0 Å in the vertical mode (Figure 2d). In contrast, in the presence of *α*_v_ *β*_8_, it exhibited three stepwise changes. At the beginning, the horizontal‐centroid distance decreased rapidly until 560 ns, which might be due to the aggregation of POPC lipids toward GO‐PEG. Then, it increased approximately till 720 ns and was finally relatively stable with almost no fluctuation (Figure 2d).

To quantify the motion of the POPC membrane, specifically the lipid aggregation toward GO‐PEG, we counted the number of POPC lipids positioned within 4 nm of GO‐PEG in both horizontal and vertical modes. Due to the increased contact area in the horizontal mode, initially about 170 POPC lipids were present; then, they became about 152 by the end of the production simulation due to the free diffusion of lipids (Figure 2e). In the vertical mode, due to the reduced contact area in the vertical mode, initially about 116 POPC lipids were present, gradually increasing to about 130 due to lipid aggregation toward GO‐PEG (Figure 2e). Meanwhile, we obtained evidence to support this simulation result by confocal imaging with isolated primary macrophages based on the intrinsically lipophilic *β*‐BODIPY 500/510 dye. Unlike control cells, the membrane of GO‐PEG‐treated macrophages contained puncta with strong enrichment for the signal intensity of the lipid‐targeting dye (Figure 2f), supporting the GO‐PEG‐induced spatial aggregation of membrane lipids.

### 2.3 Integrin *α*_v_ *β*_8_ Undergoes Conformational Changes in Response to GO‐PEG‐Mediated Lipid Aggregation

**Figure 3:**
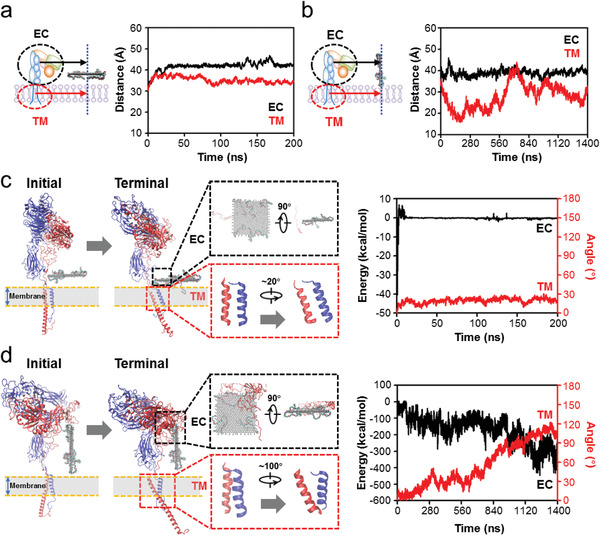
Effects of GO‐PEG on membrane protein *α*_v_ *β*_8_ in the horizontal and vertical mode of ternary model. a) Horizontal‐centroid distances between GO‐PEG and *α*_v_ *β*_8_ EC/TM domains in the horizontal mode. These distances are relatively stable. b) Horizontal‐centroid distances between GO‐PEG and *α*_v_ *β*_8_ EC/TM domains in the vertical mode. The distance between GO‐PEG and *α*_v_ *β*_8_ TM domain is also variable like that between GO‐PEG and POPC membrane lipids, as shown in Figure 2d. c) Typical structure diagrams and quantitative analysis of the interaction between GO‐PEG and *α*_v_ *β*_8_ EC/TM domains in the horizontal mode. Left panel: initial and terminal structures of production simulations, with zoomed‐in views for the detailed information between GO‐PEG and EC domains of the terminal structure (black box) and the conformation change of TM domains of the initial and terminal structures (red box); Right panel: quantitative analysis of energy between GO‐PEG and EC domains (black line) and angle between *α*_v_ and *β*_8_ subunits of TM domains (red line). d) Typical structure diagrams and quantitative analysis of the interaction between GO‐PEG and *α*_v_ *β*_8_ EC/TM domains in the vertical mode. The legend descriptions refer to those in (c). The energy and angle are variable.

Next, we carefully examined the structural characteristics of *α*_v_ *β*_8_ as a result of GO‐PEG stimulation in the ternary production simulations. We first inspected the distance between *α*_v_ *β*_8_ and GO‐PEG. In the horizontal mode, the horizontal‐centroid distance between the EC/TM domains and GO‐PEG did not change significantly, suggesting the benign stability of *α*_v_ *β*_8_ (**Figure** **3a**). In contrast, in the vertical mode, the horizontal‐centroid distance between TM domains and GO‐PEG changed in three steps (Figure 3b), similar to that between GO‐PEG and POPC membrane lipids, as shown in Figure 2d. The horizontal‐centroid distance initially decreased rapidly, which might be due to GO‐PEG‐mediated lipid aggregation to drive the proximity of the TM domain. It then increased approximately till 720 ns and finally stabilized with almost no fluctuation (Figure 3b).

We further explored the local energy and angle changes. The interaction energy between the EC domains and GO‐PEG was 0 kcal mol^−1^ in the horizontal mode, suggesting no contact between *α*_v_ *β*_8_ and GO‐PEG (Figure 3c). The TM angle, defined as the angle between initial vector and the vector from the real‐time *β*_8_ TM centroid pointing to *α*_v_ TM centroid, was ≈20°. Throughout the 200 ns ternary simulation, *α*_v_ *β*_8_ TM domains did not undergo conformational changes in the horizontal GO‐PEG interaction mode (Figure 3c). In contrast, in the vertical mode, the TM domains of the *α* and *β* subunits gradually rotated away from each other in an anticlockwise direction, particularly increasing the angle at 560 ns, and reached ≈100° by the end of the simulation (Figure 3d). This stepwise conformation change might be responsible for the increased distance from 560 to 720 ns (Figures 2d and 3b). This change in the TM domains was also confirmed by a change in the spatial arrangement of the *α*_v_ *β*_8_ EC domains as the entire EC domain was apparently brought close to the membrane lipids and GO‐PEG (Figure 3d).

**Figure 4:**
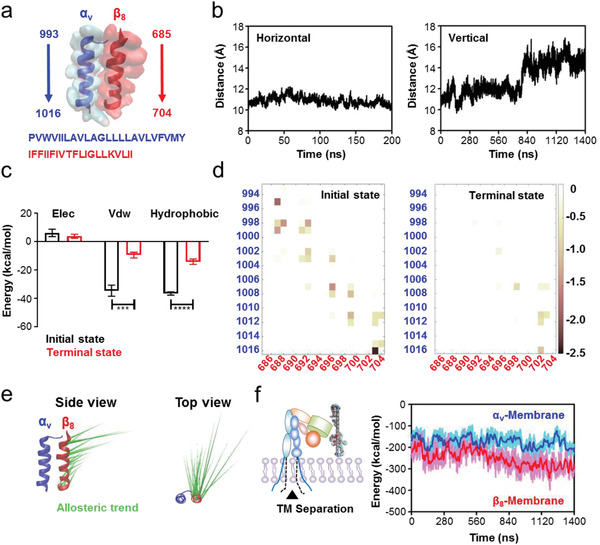
Separation of *α*_v_ *β*_8_ TM domains induced by GO‐PEG‐mediated conformational transduction. a) Structural diagrams and residues of the *α*_v_ *β*_8_ TM domains. b) Centroid distance of TM domains between the *α*_v_ and *β*_8_ subunits. Left panel: horizontal mode; Right panel: vertical mode. The increased distance in the vertical mode suggests the separation of *α*_v_ *β*_8_ TM domains. c) Free energy calculation and decomposition between the *α*_v_ and *β*_8_ subunits at the initial and terminal moments in the vertical mode. Data for initial state were obtained from three representative snapshots extracted from the first 1 ns of production simulation, and data for terminal state were obtained from three representative snapshots extracted from the final 1 ns of production simulation. Data show that van der Waals (VdW) and hydrophobic interactions are the main energy barriers for TM domain separation. d) Pairwise amino acid interaction between the *α*_v_ and *β*_8_ subunits at the initial and terminal moments in the vertical mode. Data show that the pairwise amino acid interactions are rearranged, accompanied by TM domain separation. e) PCA to assess the movement tendency of *α*_v_ *β*_8_ TM domains. *α*_v_ TM domain is overlapped and the movement tendency of *β*_8_ TM domain is visual from both the side and top view. f) Noncovalent interaction energies between the POPC membrane lipids and *α*_v_ or *β*_8_ subunit TM domains. Data show that the enhanced energy between *β*_8_ and membrane accounts for *α*_v_ *β*_8_ TM domain separation.

We focused on the conformational changes of TM domains due to the TM rotation and EC rearrangement, as TM domain separation is essential for integrin activation. The TM domains of the *α*_v_ and *β*_8_ subunits comprise residues 993–1016 and 685–784, respectively (**Figure** **4a**). We then examined the centroid distance of TM domains between the *α*_v_ and *β*_8_ subunits in horizontal and vertical modes. The distance did not change significantly in the horizontal mode (stable at around 11 Å), whereas it rapidly increased from 11 to 15 Å between 720 and 840 ns in the vertical mode (Figure 4b), indicating the occurrence of separation. The noncovalent interaction energy of TM domains between the *α*_v_ and the *β*_8_ subunits exhibited a similar trend (Figure S5, Supporting Information). Interestingly, the interaction energy change occurred after the TM domains of the *α* and *β* subunits rotated to increase their angle. The corresponding free energy analyses for the vertical mode indicated that the constraint energies between the TM domains were mainly van der Waals (VdW) and hydrophobic interactions, with weak electrostatic interactions (Figure 4c). Over the course of production simulation, both VdW and hydrophobic interaction energies significantly decreased, and the electrostatic interaction energy also decreased slightly (Figure 4c). Further pairwise amino acid interaction analyses revealed that the interactions between TM domain amino acids evolved throughout the simulation, with the original constraints being substantially weakened or even disappearing and new interactions formed by the end of the simulation (Figure 4d). However, these interaction rearrangement phenomena were not significant in the horizontal mode (Figure S6, Supporting Information). Root mean square fluctuation (RMSF) analysis also suggested that amino acids in the vertical mode were more flexible than that of horizontal mode (Figure S7, Supporting Information).

To further identify the forces driving the conformational change of *α*_v_ *β*_8_ TM domains in the vertical mode, we detected the “essential motion patterns” based on principal component analysis (PCA) of the production simulation data.^[^ ^26^ ^]^ In this approach, intuitionistic “porcupine” plots indicate intrinsic motion direction and distance. We found that *β*_8_ TM domains had the tendency to rotate away from *α*_v_ TM domains in an anticlockwise direction, and the residues approaching the EC domains had increased allosteric distance (Figure 4e). We then calculated the noncovalent interaction energies between the POPC membrane lipids and the *α*_v_ or *β*_8_ subunit TM domains. The data suggested that the interaction energy between the POPC membrane and *α*_v_ TM domains was relatively stable (about −170 kcal mol^−1^). For the *β*_8_ subunit, the interaction energy increased from about −220 to −280 kcal mol^−1^ between 560 and 840 ns (Figure 4f), suggesting a plausible physical mechanism to explain the predicted TM domain movement in the vertical mode. Collectively, our findings indicate that integrin *α*_v_ *β*_8_ undergoes a series of time‐dependent conformation changes as it adapts to its interactions with the POPC membrane and with GO‐PEG. In response to the GO‐PEG‐mediated lipid aggregation, *α*_v_ *β*_8_ TM domains approach the GO‐PEG; then they rotate away from each under the influence of membrane lipids to bring the *α*_v_ *β*_8_ EC close to GO‐PEG; finally, the *α* and *β* subunit TM domains separate.

### 2.4 Separation of TM Domains Triggers Integrin *α*_v_ *β*_8_ Activation

**Figure 5:**
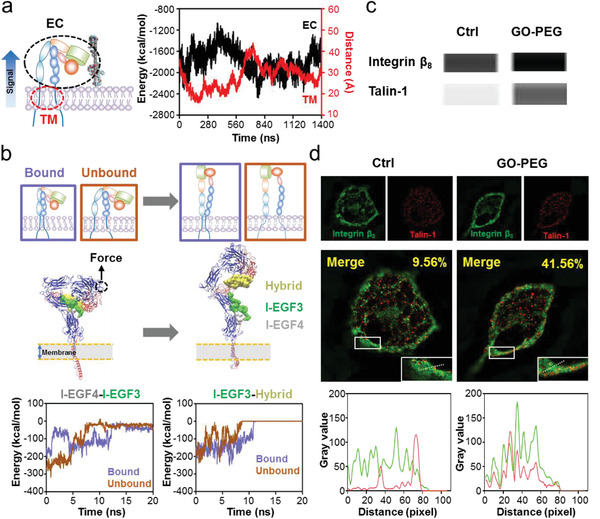
Mechanotransduction triggered by GO‐PEG‐mediated *α*_v_ *β*_8_ TM domain separation. a) Time‐dependent evolution of the interaction energy between the *α*_v_ and *β*_8_ subunit EC domains with GO‐PEG stimulation. This interaction energy displays a real‐time synchronization with the horizontal‐centroid distance between GO‐PEG and the *α*_v_ *β*_8_ TM domains in Figure 3b, which implies an outward signal transduction. b) Schematic diagrams of SMD simulations for EC domain extension starting from *α*_v_ *β*_8_ conformations with bound or unbound TM domains. The bound and unbound *α*_v_ *β*_8_ conformations were extracted from the initial structure before and from the terminal structure after GO‐PEG stimulation, respectively. The main constraints between different EC domains during the conformational extension process are also illustrated, and their time‐dependent evolutions show that unbound TM domains aid in breaking these constraints. c) Co‐immunoprecipitation of integrin *β*_8_ with talin‐1 expressed in peritoneal macrophages with and without GO‐PEG stimulation. d) Co‐localization of integrin *β*_8_ (green) and talin‐1 (red) with and without GO‐PEG stimulation. Pearson's correlation coefficient is provided in the merged image. Typical linear overlay analysis of zoomed section in white rectangular region suggests that integrin *β*_8_ and talin‐1 co‐localization increases after GO‐PEG stimulation.

Previous studies have proved that the separation of integrin TM domains is an essential procedure for bidirectional transmembrane signal transduction.^[^ ^27^, ^28^ ^]^ Accompanied by TM domain separation, integrin also undergoes other pivotal changes during its activation process, such as EC domain extension and hybrid domain swing‐out.^[^ ^12^, ^29^ ^]^ Thus, we examined the noncovalent interactions of EC domains between the *α*_v_ and *β*_8_ subunits, to determine whether GO‐PEG stimulation‐induced TM rotation and separation impacts the outward EC domain movement. This interaction changed synchronously with the horizontal‐centroid distance between the overall *α*_v_ *β*_8_ TM domains and GO‐PEG (**Figure** **5a**), suggesting a possible inside‐out signal transduction. Therefore, we extended the time scope of our simulations to investigate the consequences of EC domain extension due to GO‐PEG‐induced TM domain separation by new production simulations.

We conducted steered molecular dynamics (SMD) simulations to mimic the EC domain extension based on two initial structures. One was extracted from the initial structure of previous production simulation with bound TM domains (bound group) and the other was extracted from the terminal structure of previous production simulation with separated TM domains owing to GO‐PEG stimulation (unbound group) (Figure 5b). External force was applied to the ligand recognition site to extend the EC domains, mimicking the natural activation process. We computed the noncovalent interaction energies between different EC domains and found that the main constraints included the interactions between *α*_v_ *β*_8_ I‐EGF4 and I‐EGF3 domains and between the I‐EGF3 and hybrid domains. Their corresponding energy curves during the conformational extension process indicate that the unbound group had a rapid rupture (Figure 5b), suggesting that *α*_v_ *β*_8_ TM domain separation may accelerate EC domain extension for activation.

To validate the virtual activation of integrin *α*_v_ *β*_8_, we further studied the GO‐PEG‐mediated mechanotransduction signaling of primary macrophages. Co‐immunoprecipitation analysis indicated that GO‐PEG stimulated primary macrophages with high integrin *β*_8_ expression. Meanwhile, the combination of talin, a cytoplasmic protein that links the integrin *β* subunit to the cytoskeleton for intracellular signal transduction activity,^[^ ^30^, ^31^ ^]^ increased together with integrin *β*_8_ (Figure 5c), indirectly reflecting *α*_v_ *β*_8_ activation. Furthermore, we monitored the localization of integrin *α*_v_ *β*_8_ and talin using immunofluorescence. The co‐localization of integrin *β*_8_ (green) and talin‐1 (red) in the control macrophages not treated with GO‐PEG was significantly less than that after GO‐PEG treatment, which is confirmed by the upregulated Pearson's correlation coefficient and superior linear overlay analysis (Figure 5d and Figure S8, Supporting Information). These results experimentally demonstrated that the GO‐PEG‐induced TM domain separation triggered integrin *α*_v_ *β*_8_ activation.

### 2.5 GO‐PEG‐Induced Conformational Changes Are Not Dependent on Interaction Orientation

**Figure 6:**
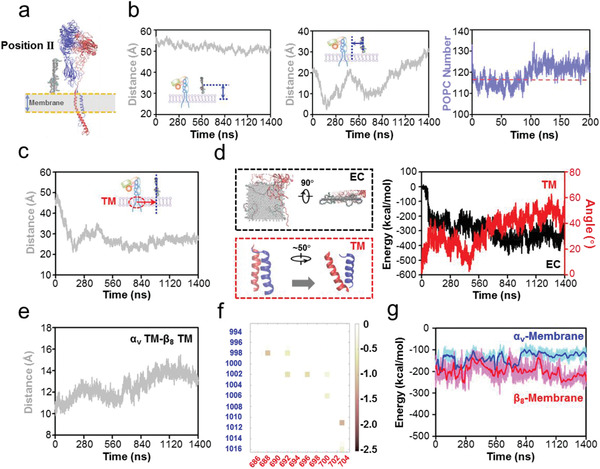
The effects of initial orientation of GO‐PEG relative to *α*_v_ *β*_8_ on *α*_v_ *β*_8_ activation in the vertical mode. a) Structural diagram of the initial ternary model. GO‐PEG is placed on the other side of *α*_v_ *β*_8_ (Position II), which is directly opposite to the position in Figure 1e. b) Effects of GO‐PEG on the POPC membrane of ternary model. Left panel: vertical‐centroid distance between GO‐PEG and POPC membrane; Middle panel: horizontal‐centroid distance between GO‐PEG and POPC membrane; Right panel: number of POPC membrane lipids within 4 nm of GO‐PEG. These data suggest that GO‐PEG mediates the horizontal motion of POPC membrane lipids. c) Effects of GO‐PEG on the motion of membrane protein *α*_v_ *β*_8_. The horizontal‐centroid distance between GO‐PEG and *α*_v_ *β*_8_ TM domains also displayed a dramatic trend in response to the motion of POPC membrane lipids. d) Effects of GO‐PEG on the EC and TM domains of *α*_v_ *β*_8_. Evolution of the interaction energy between GO‐PEG and *α*_v_ *β*_8_ EC domains and the self‐rotation angle of the TM domains also validate the previous results. e) Centroid distance of TM domains between the *α*_v_ and *β*_8_ subunits. The increased distances suggest the separation of *α*_v_ *β*_8_ TM domains. f) Pairwise amino acid interaction between the *α*_v_ and *β*_8_ subunits at the terminal moment of production simulation. The pairwise amino acid interactions rearrange in response to TM domain separation. g) Noncovalent interaction between POPC membrane lipids and *α*_v_ or *β*_8_ subunit. The enhanced energy between *β*_8_ and membrane accounts for *α*_v_ *β*_8_ TM domain separation.

As all of the performed production simulations used the GO‐PEG model in one orientation relative to the POPC membrane and *α*_v_ *β*_8_, we placed the GO‐PEG model in the opposite position (Position II) to perform production simulations in the vertical mode, to avoid undue bias for one particular spatial orientation (**Figure** **6a**). The vertical‐centroid distance between GO‐PEG and POPC membrane was stable at ≈52 Å. In contrast, the horizontal‐centroid distance between GO‐PEG and POPC membrane changed significantly over the course of this production simulation. Initially, the POPC membrane lipids were clustered to GO‐PEG, with the horizontal‐centroid distance rapidly decreasing from 22 to 4 Å in about 180 ns; then, the distance increased to 23 Å in 450 ns. The distance then fluctuated, continuously increased until the end of the simulation at 1400 ns (Figure 6b). This time‐dependent POPC membrane motion trend was similar to the one observed in the production simulation based on the initially positioned GO‐PEG model (Position I). Similar to the initial one, the simulation at Position II revealed that the changes in POPC number within 4 nm of GO‐PEG also mirrored lipid aggregation (Figure 6b). These data reproduced the lipid aggregation effects of GO‐PEG on the POPC membrane.

Next, we examined the effects of GO‐PEG‐mediated lipid aggregation on *α*_v_ *β*_8_ in the Position II simulation. We also detected the apparent GO‐PEG‐stimulated three stepwise conformational changes: the horizontal‐centroid distance between GO‐PEG and *α*_v_ *β*_8_ TM domains rapidly decreased from 49 to 22 Å by 180 ns, then gradually increased to 34 Å by about 400 ns, and finally reached a stable state after a fluctuation period (Figure 6c). The TM domains of *α*_v_ *β*_8_ also rotated, causing the TM angle to increase by ≈50°, while conformational changes in the EC domains brought them into closer contact with GO‐PEG (Figure 6d). The noncovalent interaction energy between the EC domains and GO‐PEG was improved by ≈−370 kcal mol^−1^ compared to that of the original noncontact situation (Figure 6d).

Similarly, specific comparisons between the TM domains of *α*_v_ and *β*_8_ subunits showed the mechanism of domain separation during Position II production simulation. The centroid distance between the *α*_v_ and *β*_8_ TM domains gradually expanded from 11 to 14 Å with GO‐PEG stimulation (Figure 6e), suggesting the separation of TM domains. Free energy analyses also indicated that VdW and hydrophobic interactions were major constraints between the *α*_v_ and *β*_8_ subunit TM domains, and the interactions between TM amino acids evolved throughout the simulation (Figure 6f and Figure S9, Supporting Information), consistent with the RMSF of each amino acid (Figure S10, Supporting Information). Finally, noncovalent interaction energy analyses for the POPC membrane with *α*_v_ *β*_8_ TM domains indicated that the interaction with the *α*_v_ subunit was relatively stable and the increased energy with the *β*_8_ subunit accounted for the separation of *α*_v_ *β*_8_ TM domains (Figure 6g). All these data reinforced our conclusions on GO‐PEG stimulation from the Position I simulations and further demonstrated that initial GO‐PEG orientation did not influence *α*_v_ *β*_8_ mechano‐activation.

## 3 Conclusion

**Figure 7:**
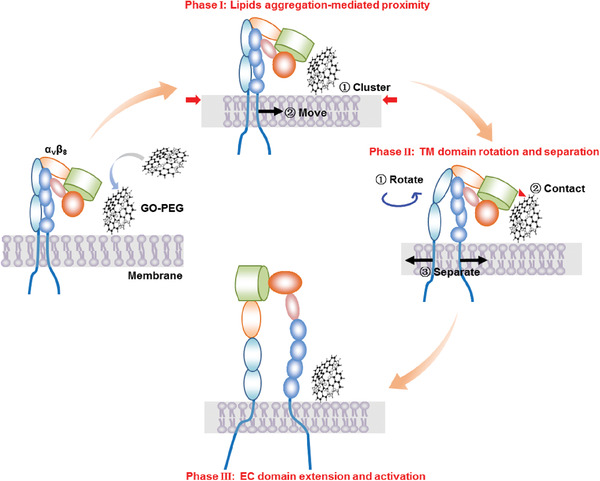
Schematic diagram of *α*_v_ *β*_8_ activation through GO‐PEG‐induced mechanotransduction on the membrane. The signal transduction pathway can be divided into three phases: Phase I: GO‐PEG mediated membrane lipid aggregation and drove the proximity of integrin *α*_v_ *β*_8_; Phase II: the TM domains of *α*_v_ *β*_8_ rotated and separated due to GO‐PEG stimulation; Phase III: the EC domains of *α*_v_ *β*_8_ responded to TM conformational changes for *α*_v_ *β*_8_ extension and activation.

In summary, we have developed a ternary simulation system consisting of a GO‐PEG nanosheet, a POPC membrane, and integrin *α*_v_ *β*_8_ to characterize nanomaterial‐mediated ligand‐independent integrin activation. This ternary simulation strategy provided an accurate model for investigating integrin mechano‐activation by nanomaterials, offering atomic scale precision, thus exceeding the resolution of previously available models. By coupling molecular dynamics simulations with a variety of experiments, we discovered that *α*_v_ *β*_8_ undergoes a series of structural changes that initiate signaling transduction events. Specifically, GO‐PEG first induced lipid aggregation‐mediated integrin proximity, followed by TM domain rotation and separation, leading to the extension and activation of EC domains (**Figure** **7**). We also observed that GO‐PEG‐mediated activation resulted in *α*_v_ *β*_8_ and talin co‐localization.

Our study offers a new perspective to examine bio–nano interfaces that mediate ligand‐independent integrin activation, and raises interesting new questions about the specific contributions of forces among particular nanomaterial sites, membrane biomolecules, and specific integrin residues. Due to the absence of an antibody to specifically monitor *α*_v_ *β*_8_ activation, cryo‐electron microscopy may help characterizing the conformation adopted in the *α*_v_ *β*_8_ activated state on the membrane after GO‐PEG stimulation. Regarding TM domain separation, free energy analysis identified several candidate residues that may mediate *α*_v_ *β*_8_ conformational changes, thus generating *α*_v_ *β*_8_ variants with targeted amino acid site mutations that can be used to experimentally assess specific functional contributions. Specifically, measuring both wild type and mutant *α*_v_ *β*_8_ variants interacting with ligands (e.g., fibronectin, vitronectin, or an RGD fragment) should enable a quantitative comparison, which precisely elucidates the particular residue‐mediated conformational changes that occur during integrin activation.

Our findings that GO‐PEG activated the potent cytokine responses of macrophage via integrin *α*_v_ *β*_8_‐mediated mechanotransduction signaling can also provide some useful guidelines for the rational design and use of nanomaterials in biomedical field. Given the capability of macrophage in antigen presentation, GO‐PEG or other *α*_v_ *β*_8_ agonists may serve as new candidates for vaccine adjuvants to enhance the immune response. Considering the important role of tumor‐associated macrophage in immunosuppressive tumor microenvironment (TME), it is also worth testifying the feasibility of utilizing GO‐PEG for ameliorating TME via macrophage polarization. However, in view of wide distribution of macrophage throughout the body, such as liver, spleen, and lung,^[^ ^32^ ^]^ attentions should be paid to the potential proinflammation in these organs. Beyond the medicine filed, coupling with other materials for GO‐PEG can also be considered, when they are practically applied in tissue engineering.^[^ ^33^, ^34^ ^]^ For example, moderate proinflammation of macrophages has been demonstrated favorable for promoting osteogenesis.^[^ ^35^ ^]^ In this aspect, surface modification of implantable devices with optimized GO‐PEG may exhibit improved outcomes for bone formation.

While this study has focused on the interfacial interactions between macrophage membranes and GO‐PEG, our findings indicate that other cell types, interaction proteins, and activation modes, as well as diverse modified and unmodified 2D nanomaterials, should be explored. Hence, GO‐PEG should be evaluated with other cell types such as other immune cells, endothelial cells, stem cells, and nerve cells, thus identifying the specific membrane protein (integrins, selectins, ion channel proteins, etc.) that interacts with GO‐PEG on the surfaces of diverse cells. Such investigations may reveal other ligand‐independent activation or manipulation of cell behavior. Additionally, our insights into bio–nano interfaces should extend from graphene‐derived nanomaterials to other 2D amphipathic PEGylated‐functionalized nanomaterials with good prospects for clinical interest, such as biodegradable PEGylated polylactic acid. Any similarities between the interaction modes and ligand‐independent or other activation mechanisms can indicate the de novo design and/or rational use of future nanomaterials to obtain the desired outcomes at diverse bio–nano interfaces.

## 4 Experimental Section

##### Materials and Chemicals

GO powder was provided by Yongjun Gao's Group of Hebei University. Methoxypolyethylene glycol (mPEG‐NH_2_, *M*_w_ = 2000) was purchased from Beijing JenKem Technology. 1‐ethyl‐3‐(3‐dimethylaminopropyl) carbodiimide (EDC) was purchased from Beijing J&K China Chemical Ltd. Sodium chloroacetate was purchased from Shanghai Sigma‐Addiction. Dulbecco's modified Eagle's medium (DMEM), penicillin/streptomycin solution, trypsin‐ethylenediaminetetraacetic acid solution, phosphate buffer saline (PBS) were all acquired from Invitrogen. Radioimmunoprecipitation assay buffer (high), 4% paraformaldehyde, and 5% bovine serum albumin (BSA) blocking buffer were purchased from Solarbio Life Science. Membrane and Cytosol Protein Extraction Kit was purchased from Beyotime. ITGB8 antibody, ITGAV antibody, GADPH antibody, and Talin‐1 antibody were all purchased from eBioscience. The chemicals above were all analytical grade. Alexa Fluor 647 goat anti‐mouse IgG (H+L), Alexa Fluor 488 goat anti‐rabbit IgG (H+L), *β*‐BODIPY 500/510 C12‐HPC, Micro BCA Protein Assay Kit, and Pierce Co‐Immunoprecipitation Kit were obtained from Thermo Scientific.

##### Nanomaterial Synthesis and Characterization

PEGylation of single‐layered GO with the lateral size of 200–300 nm was achieved following previously established method.^[^ ^20^, ^36^ ^]^ Briefly, EDC (20 × 10^−3^ m) was added into GO suspension (500 µg mL^−1^), sonicated for 15 min, and then reacted overnight with addition of mPEG‐NH_2_ (10 mg mL^−1^). The products were collected by centrifugation at 11 000 *g* after repeated washing with deionized water. The morphology of GO/GO‐PEG was imaged by atomic force microscope (Bruker) and analyzed by software NanoScope Analysis 1.80 (Bruker). More detailed preparation method and characterizations of GO/GO‐PEG are included in Supporting Information.

##### Animals and Cells

C57BL/6N mice, 6–8 weeks of age, were obtained from Vital River Laboratories (Beijing, China). This study was performed in strict accordance with the Regulations for the Care and Use of Laboratory Animals and Guideline for Ethical Review of Animal (China, GB/T 35892‐2018). All animal experiments were reviewed and approved by the Animal Ethics Committee of the Institute of Process Engineering (approval ID: IPEAECA2019111). Following a typical protocol,^[^ ^37^ ^]^ primary mouse macrophages were obtained from stimulated C57BL/6N mice. These macrophages were cultured with DMEM cell culture medium added with penicillin (100 U mL^–1^), streptomycin (100 U mL^–1^), and 10% fetal bovine serum at 37 °C in a humidified incubator containing 5% CO_2_.

##### Integrin *β*_8_ Detection

The primary macrophages were incubated with GO/GO‐PEG at 10 µg mL^−1^ for 24 h, using PBS treatment as control group. Cells were fixed with 4% paraformaldehyde for 30 min, blocked with 1% BSA blocking buffer for 30 min, and then incubated with ITGB8 antibody (1:200) as primary antibodies at 4 °C overnight and anti‐rabbit IgG conjugated with Alexa Fluor 488 dyes at room temperature for 1 h. The nucleus was stained by 4′,6‐diamidino‐2‐phenylindole. Integrin *β*_8_ imaging was performed using confocal laser scanning microscopy (Nikon), and fluorescence intensity was detected by flow cytometry (Beckman).

##### Preparation of Primary Macrophage Cell Membrane

The cell membrane was prepared according to the method reported previously.^[^ ^38^, ^39^, ^40^ ^]^ The primary macrophage cells were washed and resuspended in PBS buffer solution supplemented with a protease inhibitor cocktail. The macrophage suspension was destructed by IKAT18 basic ULTRA‐TURRAX (IKA, Germany) on the ice and the consequent cellular membrane fragments were purified by discontinuous sucrose density gradient ultracentrifugation at 4 °C. The obtained membrane fragments were collected for interface mechanics analysis.

##### Interfacial Interaction between GO‐PEG and Cell Membrane

Interfacial interaction between GO‐PEG and cell membrane was characterized by QCM (Q‐Sense). 100 µL cell membrane fragments were spin‐coated onto the Au chip, and GO‐PEG solution (0.2 mg mL^−1^) was pumped into the cell with the flow velocity of 50 µL min^−1^ for interaction with the cell membrane. The real‐time interaction was reflected by quality change on the chip, which was converted into frequency change of the output electrical signal. After the curve reached a relatively stable level, the deionized water was pumped and rinsed the unbound GO‐PEG.

##### TEM Characterization of Cell Membrane Interacting with GO‐PEG

The primary macrophages were seeded (1 × 10^5^ mL^–1^) in a petri dish and incubated with or without GO‐PEG at 10 µg mL^−1^ for 24 h. The culture medium was then washed twice with cold PBS. Samples were fixed in 2.5% glutaraldehyde for 2 h at room temperature, then rinsed with 0.1 m PB thrice and kept at 4 °C. Afterward, samples were post‐fixed, serially dehydrated with ethanol, and embedded in epon. Finally, serial sections were cut on a Reichert Ultracut microtome (Leica), and electron micrographs were taken using a JSM‐1400 Flash transmission electron microscope. TEM images with low magnification were captured by side‐mounted PHURONA TEM camera (EMSIS). Contact angels between GO‐PEG and membrane were measured with the three‐point angle in the software Radius 2.0.

##### Lipid Distribution

The primary macrophages were seeded (1 × 10^5^ mL^–1^) in a petri dish and incubated with or without GO‐PEG at 10 µg mL^−1^ for 24 h. The phospholipid bilayer of living cells was stained with fluorescent BODIPY. Lipid variation was tracked and the intensity distribution was analyzed using Ti2 inverted structured illumination microscopy (Nikon).

##### Co‐Immunoprecipitation of Integrin *β*_8_ and Talin‐1

The primary macrophages were seeded in plate and incubated with GO‐PEG at 10 µg mL^−1^ for 24 h. Cells were then washed twice by ice‐cold PBS. Next, cells were lysed in ice‐cold IP lysis/wash buffer and collected by centrifugation at 4 °C. The protein concentration of the collected supernatant was measured by BCA assay kit. Briefly, cell lysates were incubated with integrin *β*_8_ antibody at 4 °C and treated following the instruction of Pierce Co‐Immunoprecipitation Kit. The fluid‐through of protein complex was analyzed with talin‐1 antibody using ProteinSimple Wes Capillary Western Blot analyzer. An equal amount of proteins was diluted 1:4 with sample buffer (ProteinSimple) and the quantification was performed using a 66–440 kDa 25‐lane plate (SM‐W008; ProteinSimple) in Wes according to the manufacturer's instructions.

##### Co‐Localization of Integrin *β*_8_ and Talin‐1

Immunofluorescence experiments were conducted using ITGB8 antibody (1:200) and talin‐1 antibody (1:200) for primary antibodies and anti‐rabbit IgG conjugated with Alexa Fluor 488 and anti‐mouse IgG with Alexa Fluor 647 dyes for confocal laser scanning microscopic imaging. The co‐localization images and Pearson's correlation coefficient of co‐localization were achieved by N‐SIM super resolution microscope (Nikon). The images were then analyzed by ImageJ software (version 1.52a).

##### Equilibrium and Production Simulation

The NAMD program with CHARMM27 all‐atom force field was used for the simulations. A Langevin integrator (310 K; 1 atm), an integration time step of 2 fs, and periodic boundary conditions were applied in the simulations. A smooth (10–12 Å) cutoff and the particle mesh Ewald method were employed to calculate van der Waals forces and full electrostatic interactions, respectively. More detailed information about molecular dynamics simulations is provided in the Supporting Information.

##### Statistical Analysis

All the data were presented as means ± s.d. Unpaired student's *t*‐test (two‐tailed) was used for comparison between two groups. Statistical significance between multiple groups in Figure S3 in the Supporting Information was calculated using one‐way analysis of variance. Statistical significance was set at ^*^ *p* < 0.05, ^**^ *p* < 0.01, ^***^ *p* < 0.001, and ^****^ *p* < 0.0001. All analyses were performed with GraphPad Prism software (version 9.0.0).

## Conflict of Interest

The authors declare no conflict of interest.

## Supporting information

Supporting Information

## Data Availability

The data that support the findings of this study are available from the corresponding author upon reasonable request.
