# Supplementary material for: A High‐Resolution Ternary Model Demonstrates How PEGylated 2D Nanomaterial Stimulates Integrin α v β 8 on Cell Membrane
Source: Adv Sci (Weinh). 2021 Mar 23;8(11):2004506. doi: 10.1002/advs.202004506 (PMC8188183; doi:10.1002/advs.202004506)
Supplement: Supplementary file 1 — Supporting Information [file ADVS-8-2004506-s001.pdf]

© 2021 Wiley-VCH GmbH

ADVANCED SCIENCE
Open Access

Supporting Information

for *Adv. Sci.,* DOI: 10.1002/advs.202004506

A High-Resolution Ternary Model Demonstrates How PEGylated 2D Nanomaterial Stimulates Integrin $\alpha_v\beta_8$ on Cell Membrane

*Xiao Zhang, Zhaowen Ding, Guanghui Ma, and Wei Wei**

Supplementary Experimental Section/Methods

Preparation of graphene oxide (GO): GO was made by a modified Hummers method.[1] To avoid heavy metal (Mn) contamination in GO product, 3% H_2O_2 was used for reduction of residual $KMnO_4$ and MnO_2 to manganese sulphate salts. These sulphate salts were removed by rinsing with 5% HCl, repeatedly washing with deionized water by centrifugation. Through the above purification process, neutral GO was finally allowed to be used into animals and cells.

Synthesis of PEGylated GO (GO-PEG): The single layered GO with size of 200-300 nm was achieved by sonication for 2 h in the ultrasonic tank and separated from GO solution by 90000-110000 g centrifugation (Beckman) for 20 min. Next, NaOH (8 g) and chloroacetic acid (11.658 g) were added to GO aqueous suspension (5 mL, with concentration of 100 μg/mL) for conversion hydroxyl and epoxide groups to carboxyl groups.[2] After 70 min stirring at room temperature, the resulting solution was neutralized with 6 N HCl, purified by repeating rinsing and centrifugation, and diluted to 500 μg/mL with deionized water. EDC was then added to reach 20 mM and the mixture was sonicated for 15 min, followed by addition of mPEG-NH_2 to reach 10 mg/mL and stirred for 12 h. The GO-PEG was finally obtained by repeating rinsing and centrifugation.

Characterization of GO/GO-PEG: The GO powder before and after purification was mixed with spectral pure boronic acid and then pressed into tablet. The tablet was detected by X-ray fluorescence (PANalytical B.V., AXIOS) to conduct the element analysis, and the Mn content was determined (Table S1, Supporting Information).

Morphology characterization was performed on Dimension FastScan Bio atomic force microscopy (AFM, Bruker) in the fastscan mode in air at room temperature. The GO/GO-PEG for AFM image was prepared by depositing a drop of diluted solution onto a mica plate (Figure S1a, Supporting Information). Anhydrous ethanol was used for diluent. In addition, the surface dimension and thickness of GO/GO-PEG were measured and analyzed by software NanoScope Analysis 1.80 (Bruker) (Figure S1b, Supporting Information). The zeta potential analysis of the GO/GO-PEG in an aqueous dispersion were performed on Malvern Instruments (Figure S1c, Supporting Information). The GO-PEG solution obtained after a series of synthesis procedures was dried into powder, which was further pulverized with chromatographically pure KBr. Next, a pressure of approximate 2 tons was applied to form transparent pellet. The Fourier transform infrared spectra of these pellets (Figure S1d, Supporting Information) were measured with KBr as background by Fourier transform infrared spectrometer (NICOLET iS 50).

Primary macrophage stimulation: For primary macrophage stimulation, each mouse was intraperitoneally injected with 1 mL thioglycollate solution (1 g tryptone, 0.5 g sodium chloride, 0.3 g beef extract and 6 g soluble starch were dissolved and boiled in 100 mL sterilized water) and kept for 3 days. Each mouse was intraperitoneally injected with 5 mL PBS and gently massaged. Next, the abdomen fluid was collected and washed twice with PBS by centrifugation. The centrifugal sedimentation was resuspended with cell culture medium and cultured in cell plate.

Western Blotting: The expression of integrin β_8, integrin α_v and GADPH protein were

evaluated by ProteinSimple Wes^{TM} Capillary Western Blot analyzer. The total membrane protein of primary macrophages was extracted using Membrane and Cytosol Protein Extraction Kit. Total protein was extracted by RIPA lysis buffer and quantified using the BCA assay kit. An equal amount of proteins was diluted 1:4 with sample buffer (ProteinSimple) and the quantification was performed using a 12-230 kDa 25-lane plate (PS-MK15; ProteinSimple) in Wes according to the manufacturer's instructions.

GO-PEG model construction and equilibrium simulation: A 5 nm × 5 nm bare graphene sheet was first created using VMD's Nanotube Builder plugin.[3] Then hydroxyl, carboxyl, and epoxide groups were added to the faces and edges of the graphene sheet to construct GO nanosheet. GO nanosheet was based on the Lerf-Klinowski structural model with a molecular formula of $C_{10}O_1(OH)_1(COOH)_{0.5}$, which represented typical outcomes from standard oxidation processes.[4] Extended polyethylene glycol chain configurations were generated according to our previous reported work.[5] Briefly, single PEG chain consisted of 15 ethylene glycol monomers (from RCSB PDB entry), a methylated terminus, and an amide linkage to the GO nanosheet, resulting in an approximate molecular weight of 762 amu/polymer. Six carboxyl groups were replaced with the amide linkages on each polymer, covalently attaching the PEG chains to the GO nanosheet under consideration.

Next, GO-PEG model was solvated in a 20 × 20 × 10 nm box of TIP3P molecules and ionized with 0.15 M sodium and chloride ions for equilibrium simulation. Stringent harmonic restraints were placed on both the graphene nanosheet and terminal carbon

atoms attached to oxygen-containing functional groups and PEG chains. The NAMD program with CHARMM27 all-atom force field was used for the simulations.[6] An integration time step of 2 fs and periodic boundary conditions were applied in the simulations. A smooth (10-12 Å) cutoff and the particle mesh Ewald method were employed to calculate van der Waals forces and full electrostatic interactions, respectively. Simulation trajectories were extended until the PEG adsorption process was deemed to be complete and an equilibrated structure of GO-PEG model was extracted for use in subsequent simulations.

Membrane model construction and equilibrium simulation: A 12 nm × 12 nm and an 8 nm × 8 nm segment of a pre-equilibrated POPC lipid bilayer were generated using the Membrane Builder plugin in VMD for the simulation with or without $\alpha_v\beta_8$, respectively. Additional equilibration simulations were conducted for thoroughness. After solvation in TIP3P and the deletion of water molecules in the transmembrane region, lipid tails were melted (with head groups restrained) for 25 ns. The entire system was then equilibrated without restraint for an additional 25 ns. Force field parameters were directly based on CHARMM27 all-atom force field. An equilibrated membrane configuration was extracted for use in subsequent simulations.

$\alpha_v\beta_8$ model construction and equilibrium simulation: Integrin $\alpha_v\beta_8$ was constructed using a standard homology modeling method by online Swiss-Model plugin.[7] Briefly, the extracellular domains were constructed according to the $\alpha_v\beta_3$ extracellular domains (PDB ID: 3IJE), and the transmembrane and intracellular domains were constructed according to $\alpha_{IIb}\beta_3$ transmembrane-cytoplasmic heterocomplex (PDB ID: 2KNC). The

integrated $\alpha_v\beta_8$ was first experienced an equilibrium simulation for structural relaxation and stability (approximately 50 ns), then assembled with the equilibrated POPC membrane according to the VMD tutorial. Another equilibrium simulation (approximate 300 ns) was perform for structural stability. Force field parameters were directly based on CHARMM27 all-atom force field. An equilibrated membrane configuration was extracted for use in production simulations.

Production simulations: The ternary model was constructed by integrating above equilibrated models. According to the TEM data, the equilibrated GO-PEG was placed in either a horizontal or vertical configuration with 1 nm from the membrane surface. After solvation with TIP3P, deletion of water molecules in the transmembrane region, and ionization, production runs were conducted under the same restraints used in free GO-PEG equilibrium simulations, employing the same force field and simulation parameters described previously. All systems were simulated until satisfactory convergence was evident.

Steered molecular dynamics (SMD) simulations: SMD simulations were conducted based on initial structures wherein the transmembrane domains were bound (bound group) and a terminal structure wherein the transmembrane domains were separated after above production simulations (unbound group). The atoms of transmembrane domains were fixed, and a 400 pN force vertically upwards was applied on the CA atom of W144 of α_v subunit to extend the conformation of $\alpha_v\beta_8$, until the completely extended conformation.

Simulation analysis: Most of the simulated parameters, such as RMSD, distance, energy,

were analyzed and acquired using built-in Tcl scripts of VMD. The script file can be written in Tcl code as needed, and then executed in VMD. Free energy analysis and decomposition were carried out by MM/PBSA module of AmberTools.

Table S1. **Heavy metal contents of graphene oxide (GO) samples before and after purification process.** The decreased content of Mn in GO powder after purification demonstrated the samples avoided Mn contamination.

GO	Mn Concentration (%)
Before purification	1.19507
After purification	0.00360

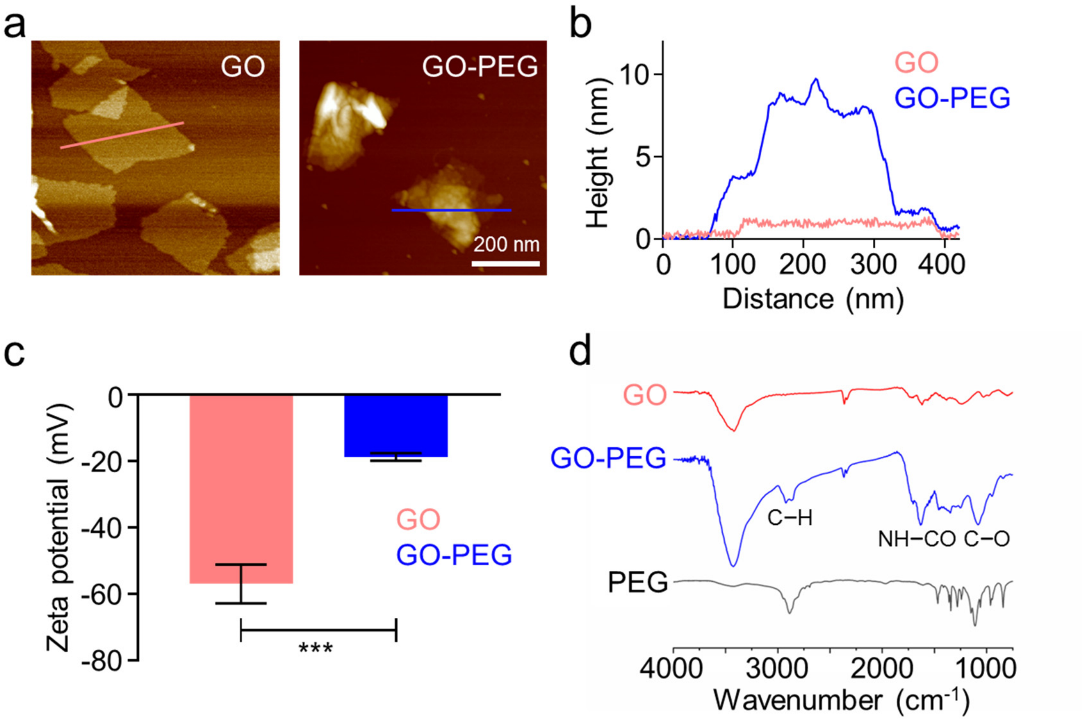

Figure S1. Characterization of PEGylated graphene oxide (GO-PEG).

(**a**) Typical AFM image of prepared GO-PEG. The size is about 200-300 nm, and GO was used as a control.

(**b**) Linear thickness analysis of GO-PEG in AFM image of (a). The thickness of GO-PEG is about 8 nm as the result of PEG modification, with GO thickness being about 1 nm.

(**c**) Zeta potential analysis of the GO-PEG in an aqueous dispersion. GO-PEG exhibited a weaker electronegativity due to PEG passivation in comparison with GO.

(**d**) Fourier transform infrared spectra of PEG, GO and GO-PEG. The successful PEGylated modification on GO was confirmed by C−H stretching vibration (~2900 cm^{-1}), C−O stretching vibration (~1100 cm^{-1}), and NH−CO stretching vibration (~1640 cm^{-1}) sourced from methoxypolyethylene glycol (mPEG-NH_2) molecules.

All the data suggested that GO-PEG was successfully prepared and could be applied in the subsequent experiments.

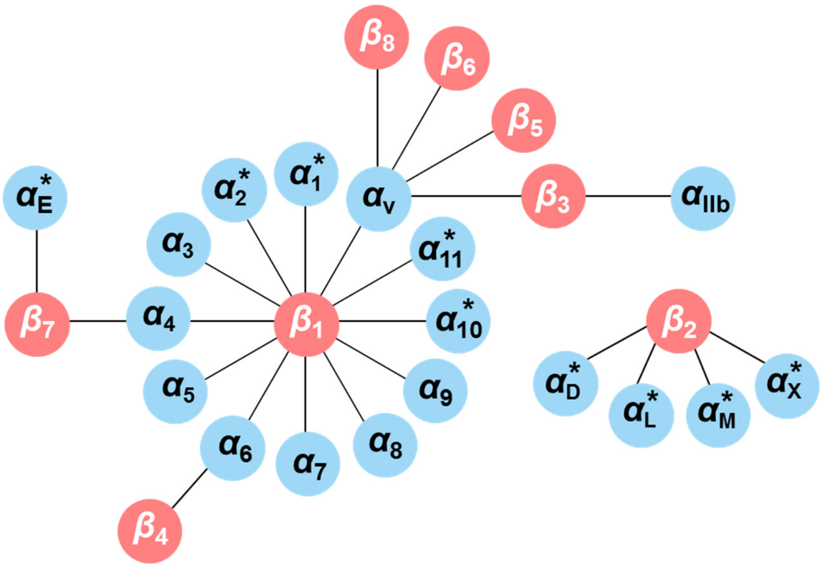

Figure S2. Integrin family members. The 24 integrin heterodimers comprised of α subunit (blue circle) and β subunit (red circle) were listed, and α subunits with α I domains were asterisked. Especially, β_8 subunit was a unique part of integrin $\alpha_v\beta_8$.

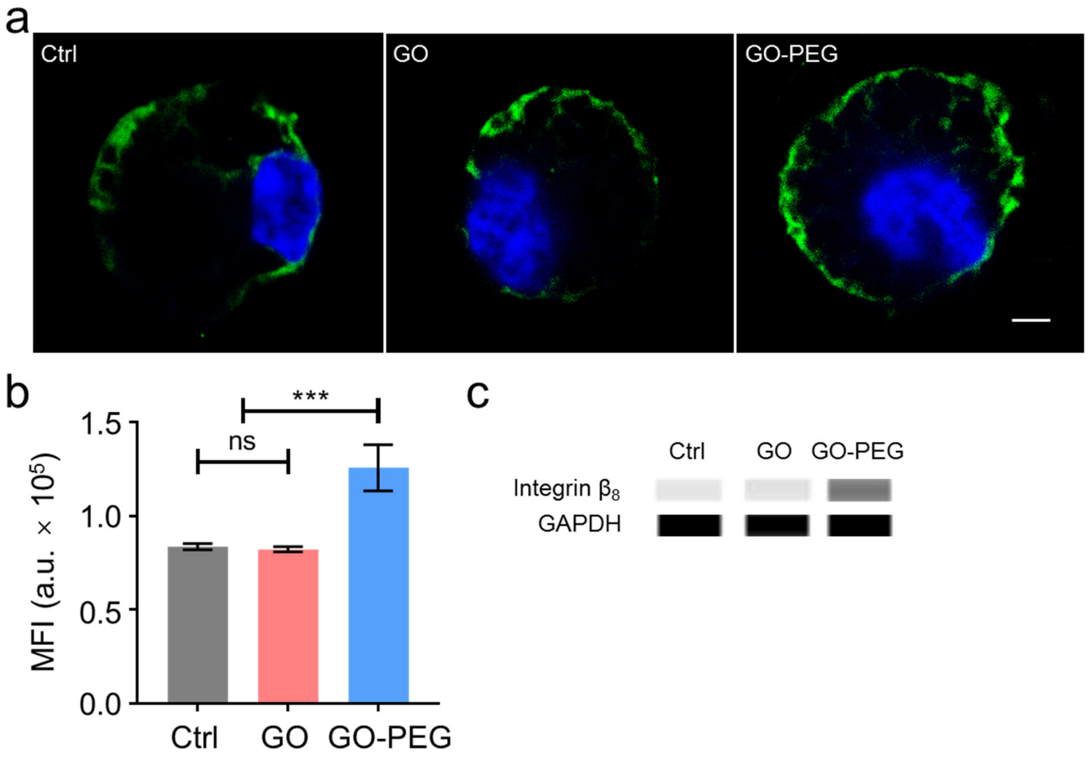

Figure S3. The expression of integrin β_8 on primary macrophages under graphene oxide (GO) and PEGylated GO (GO-PEG) stimulation.

(**a**) Confocal laser scanning microscope images of integrin β_8 (green) on primary macrophages under GO and GO-PEG stimulation, using PBS treatment as control group. Scale bar: 2 μm.

(**b**) Mean fluorescence intensity (MFI) of integrin β_8 on primary macrophages under GO and GO-PEG stimulation by flow cytometry (n = 3).

(**c**) Western blotting analysis of integrin β_8 expression levels on primary macrophages under GO and GO-PEG stimulation by ProteinSimple Wes^{TM} Capillary Western Blot analyzer. Glyceraldehyde-3-phosphate dehydrogenase (GAPDH) was used as a housekeeping gene.

These data indicated that GO-PEG induced most level of integrin β_8 than those of GO and control group.

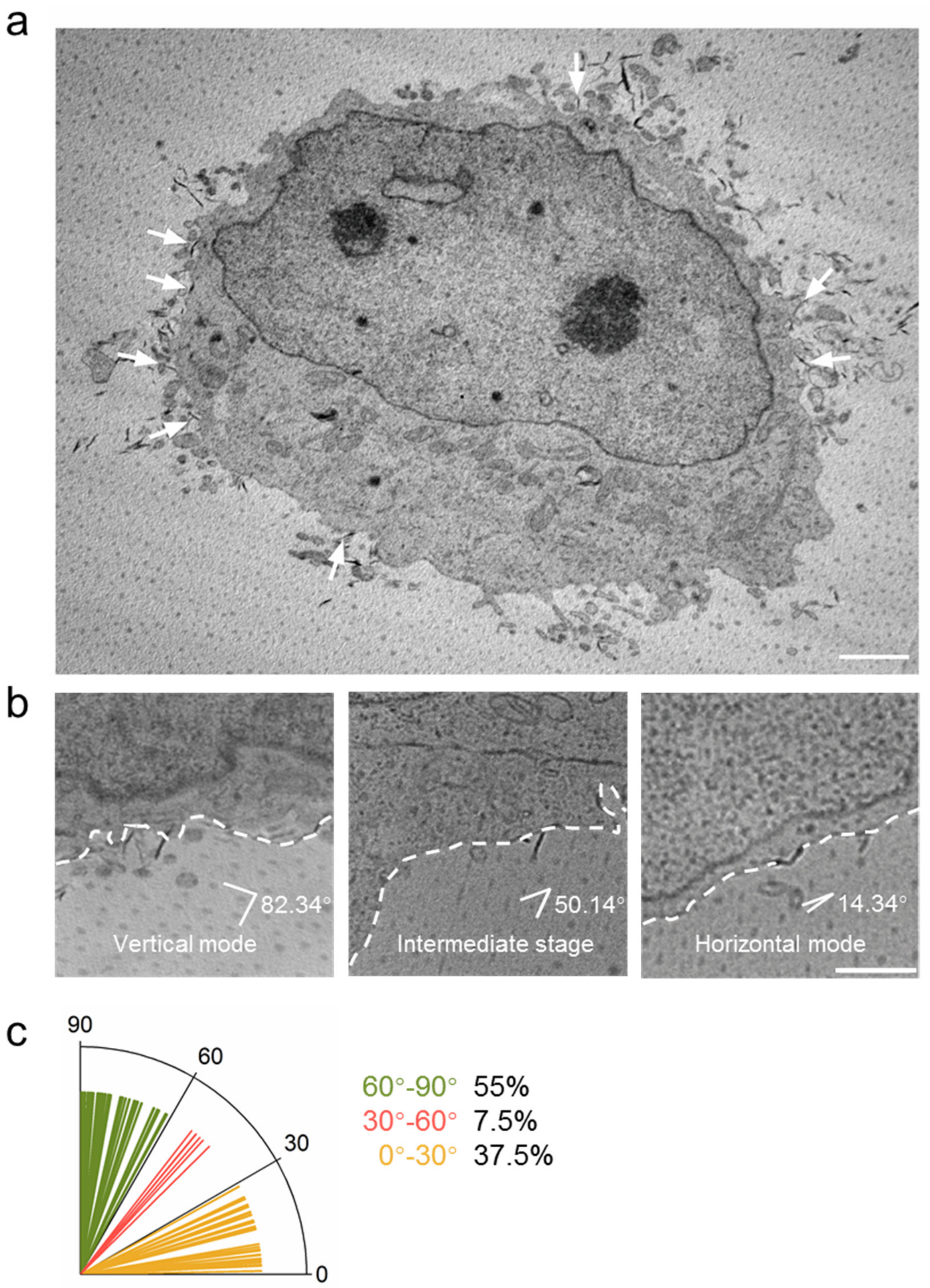

Figure S4. Interfacial interaction between PEGylated graphene oxide (GO-PEG) and macrophage membrane.

(**a**) A bird's-eye view of the interaction between GO-PEG and macrophage membrane by TEM. Interfacial interactions were indicated by the arrows. Scale bar: 2 μm.

(**b**) Representative images for the classification of contact angles between GO-PEG and macrophage membrane. Scale bar: 1 μm.

(**c**) Quantification of contact angles between GO-PEG and macrophage membrane. These data were consistent with the previously reported theoretical analysis[8]: during the interaction between 2D nanomaterial and cell membrane, the 2D nanomaterial would spontaneously tend to the orientation with a minimum energy state, that is horizontal/vertical mode. Such a domination thus verified the rational classification of horizontal mode and vertical mode for our ternary model construction and subsequent simulation.

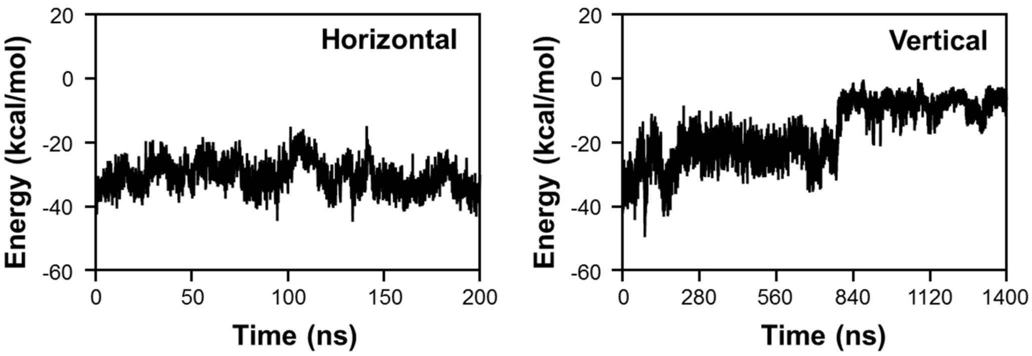

Figure S5. Non-covalent interaction energy of TM domains between the α_v subunit and the β_8 subunit during the production simulation. In the horizontal mode, TM domains of α_v subunit and β_8 subunit maintained a stable state; in the vertical mode, TM domains of α_v subunit and β_8 subunit separated from each other, with the energy almost disappeared.

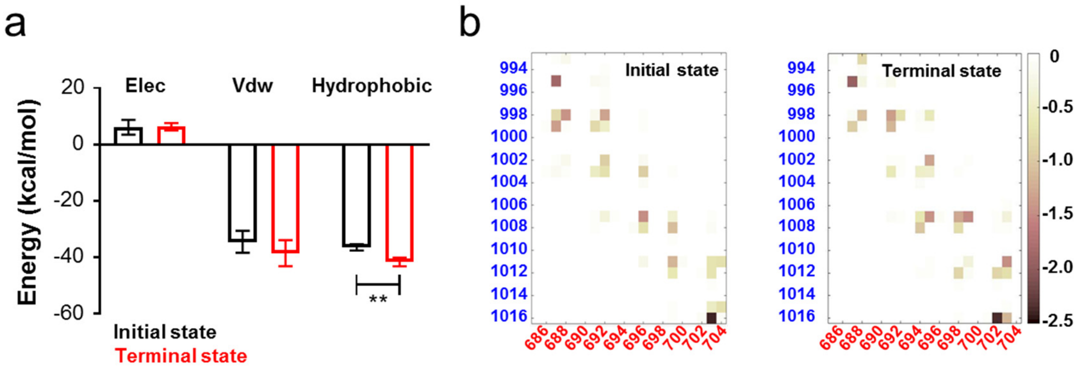

Figure S6. Free energy analysis of TM domains between α_v subunit and β_8 subunit under PEGylated graphene oxide (GO-PEG) stimulation in the horizontal mode.

(**a**) Free energy calculation and decomposition of TM domains between the α_v subunit and the β_8 subunit at the initial and terminal moments in the horizontal mode. Data for initial state were obtained from three representative snapshots extracted from the first 1 ns of production simulation, and data for terminal state were obtained from three representative snapshots extracted from the final 1 ns of production simulation.

(**b**) Amino acid pairwise interaction of TM domains between the α_v subunit and the β_8 subunit at the initial and terminal moments in the horizontal mode.

Data showed that the total free energy and amino acid pairwise interaction of TM domains between α_v subunit and β_8 subunit had almost no change under GO-PEG stimulation in the horizontal mode.

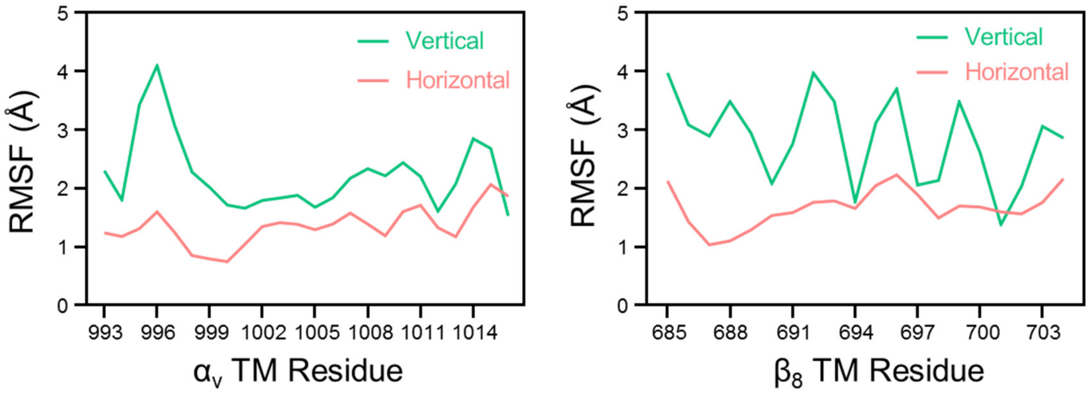

Figure S7. Root mean square fluctuation (RMSF) analysis for the transmembrane residues of integrin $\alpha_v\beta_8$ in the horizontal and vertical interaction modes of the Position I. Compared to the horizontal mode, residues in the vertical mode were more flexible and experienced more substantial conformational changes.

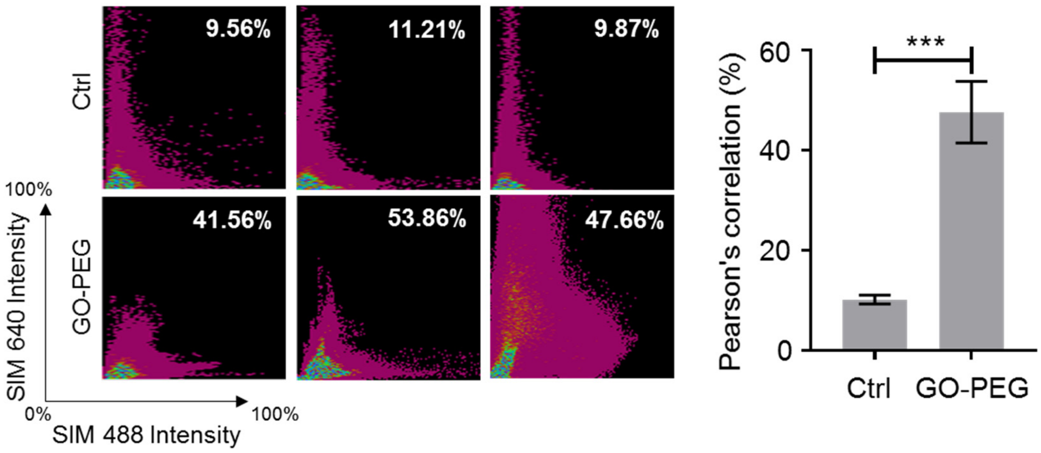

Figure S8. Colocalization of integrin β$_8$ and talin-1 with and without PEGylated graphene oxide (GO-PEG) stimulation. Integrin β$_8$ was detected via SIM 488 laser and talin-1 was detected via SIM 640 laser. Pearson's correlation coefficient increased from 10.21 ± 0.88% and 47.69 ± 6.15% after GO-PEG stimulation (n = 3).

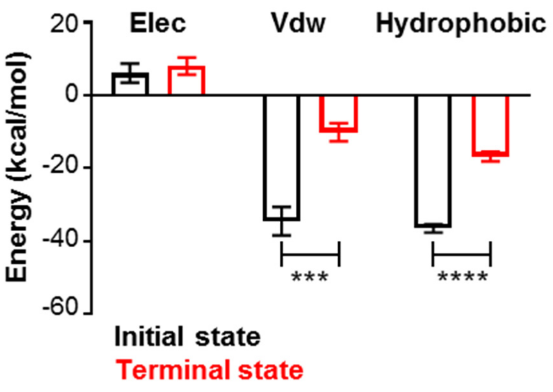

Figure S9. Free energy calculation and decomposition of TM domains between α_v subunit and β_8 subunit at the initial and terminal moments in the vertical mode under a relative orientation of PEGylated graphene oxide (GO-PEG) (Position II). Data for initial state were obtained from three representative snapshots extracted from the first 1 ns of production simulation, and data for terminal state were obtained from three representative snapshots extracted from the final 1 ns of production simulation. Data showed that the van der Waals (VdW) and hydrophobic interactions also exhibited a significantly decrease due to GO-PEG stimulation, just as the original orientation (Position I).

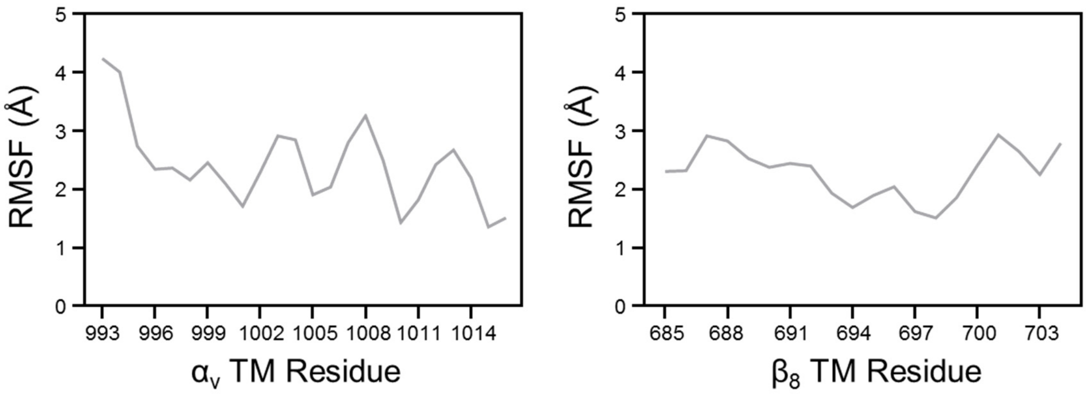

Figure S10. Root mean square fluctuation (RMSF) analysis for transmembrane residues of integrin $\alpha_v\beta_8$ in the vertical interaction modes of the Position II. The RMSF value of amino acids were consistent with their pairwise interaction energy change after the TM domains separation under PEGylated graphene oxide (GO-PEG) stimulation.

Supplementary references

[1] D. C. Marcano, D. V. Kosynkin, J. M. Berlin, A. Sinitskii, Z. Sun, A. S. Slesarev, L. B. Alemany, W. Lu, J. M. Tour, *ACS Nano* **2018**, *12*, 2078.

[2] H. Wang, Q. Zhang, X. Chu, T. Chen, J. Ge, R. Yu, *Angew. Chem. Int. Edit.* **2011**, *50*, 7065.

[3] W. Humphrey, A. Dalke, K. Schulten, *J. Mol. Graph.* **1996**, *14*, 33.

[4] H. He, J. Klinowski, M. Forster, A. Lerf, *Chem. Phys. Lett.* **1998**, *287*, 53.

[5] N. Luo, J. K. Weber, S. Wang, B. Luan, H. Yue, X. Xi, J. Du, Z. Yang, W. Wei, R. Zhou, G. Ma, *Nat. Commun.* **2017**, *8*, 14537.

[6] J. C. Phillips, R. Braun, W. Wang, J. Gumbart, E. Tajkhorshid, E. Villa, C. Chipot, R. D. Skeel, L. Kalé, K. Schulten, *J. Comput. Chem.* **2005**, *26*, 1781.

[7] A. Waterhouse, M. Bertoni, S. Bienert, G. Studer, G. Tauriello, R. Gumienny, F. T. Heer, T. A. P. de Beer, C. Rempfer, L. Bordoli, R. Lepore, T. Schwede, *Nucleic Acids Res.* **2018**, *46*, W296.

[8] X. Yi, H. Gao, *Nanoscale* **2015**, *7*, 5457.
